# Supplementary figures and images for: Foraging animals use dynamic Bayesian updating to model meta-uncertainty in environment representations
Source: PLoS Comput Biol. 2025 Apr 30;21(4):e1012989. doi: 10.1371/journal.pcbi.1012989 (PMC12068741; doi:10.1371/journal.pcbi.1012989)

$RSI = 0.05$

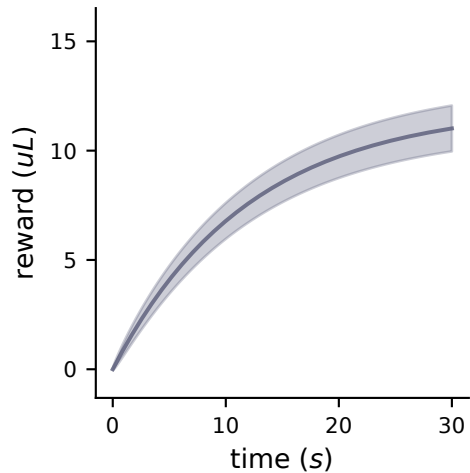

$RSI = 0.50$

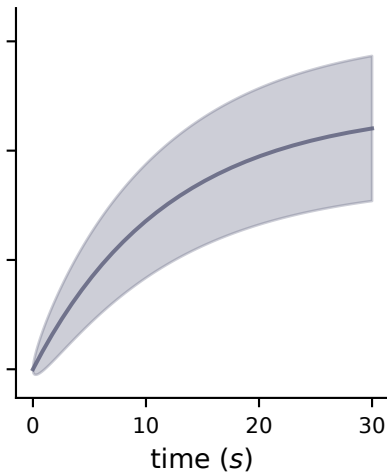

$RSI = 1.00$

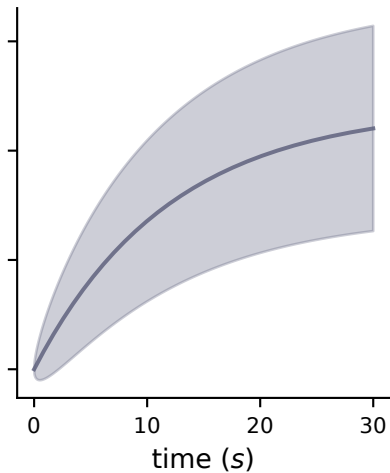

Supplement: S1 Fig — (top) Cumulative reward functions for environments with τ=12.0 and a low (left), moderate (center), or high (right) level of stochasticity in the reward dynamics, shown as mean (V0Λ(t), solid curve) ± standard deviation (V02Λ(t), shaded area). (bottom) Three example reward sequences for each level of stochasticity. (PDF) [file pcbi.1012989.s001.pdf]

**a**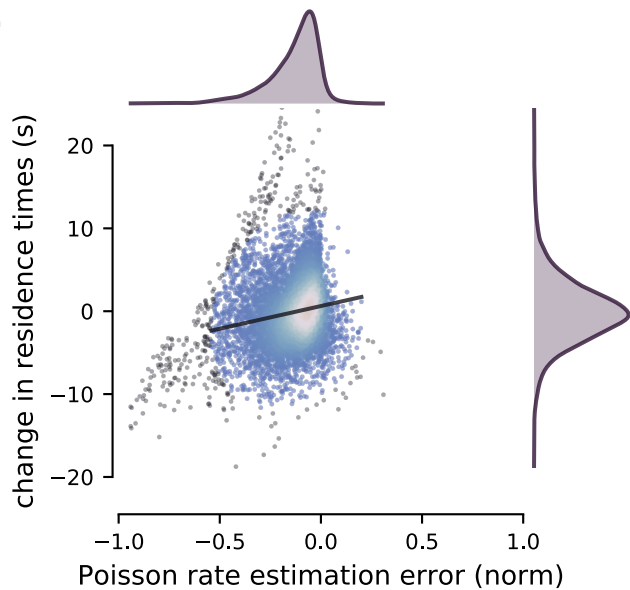**b**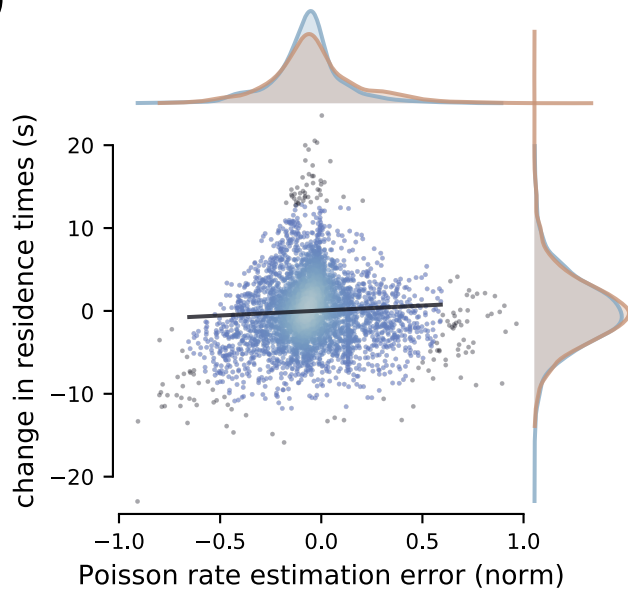

Supplement: S2 Fig — Within sessions, residence times were shuffled across patches prior to calculating to rate estimation error at patch-leaving and the change in residence time relative to the session average. The scatter plot, regression line, and marginal distributions were then calculated from the shuffled data as in Fig 4B–4C for environments with (A) low (RSI = 0.05) and (B) moderate to high (RSI∈[0.5,1.0]) stochasticity. (PDF) [file pcbi.1012989.s002.pdf]

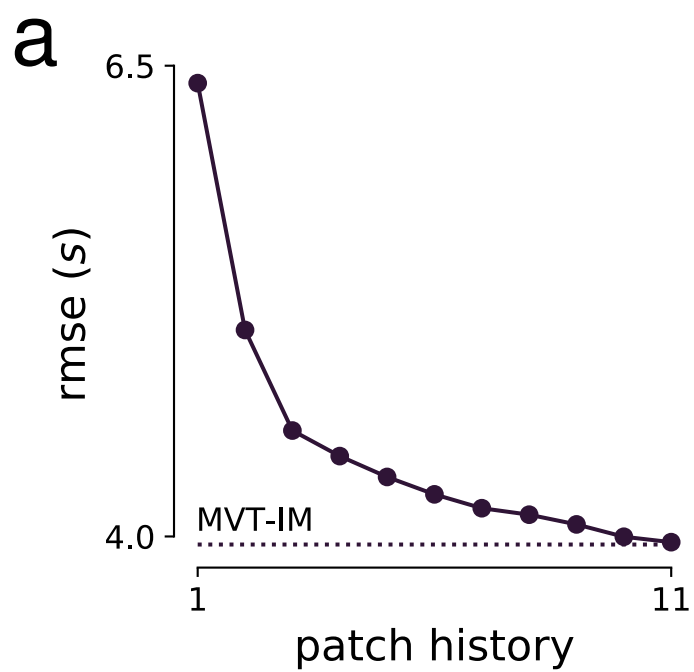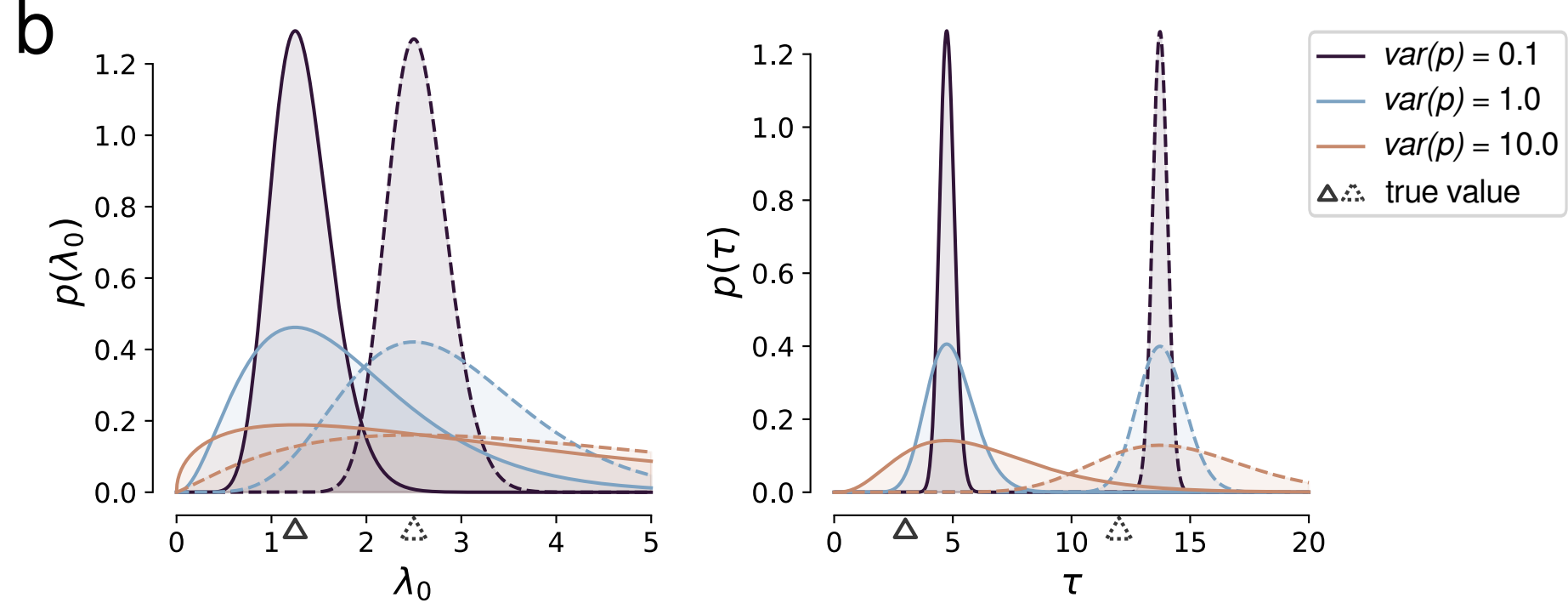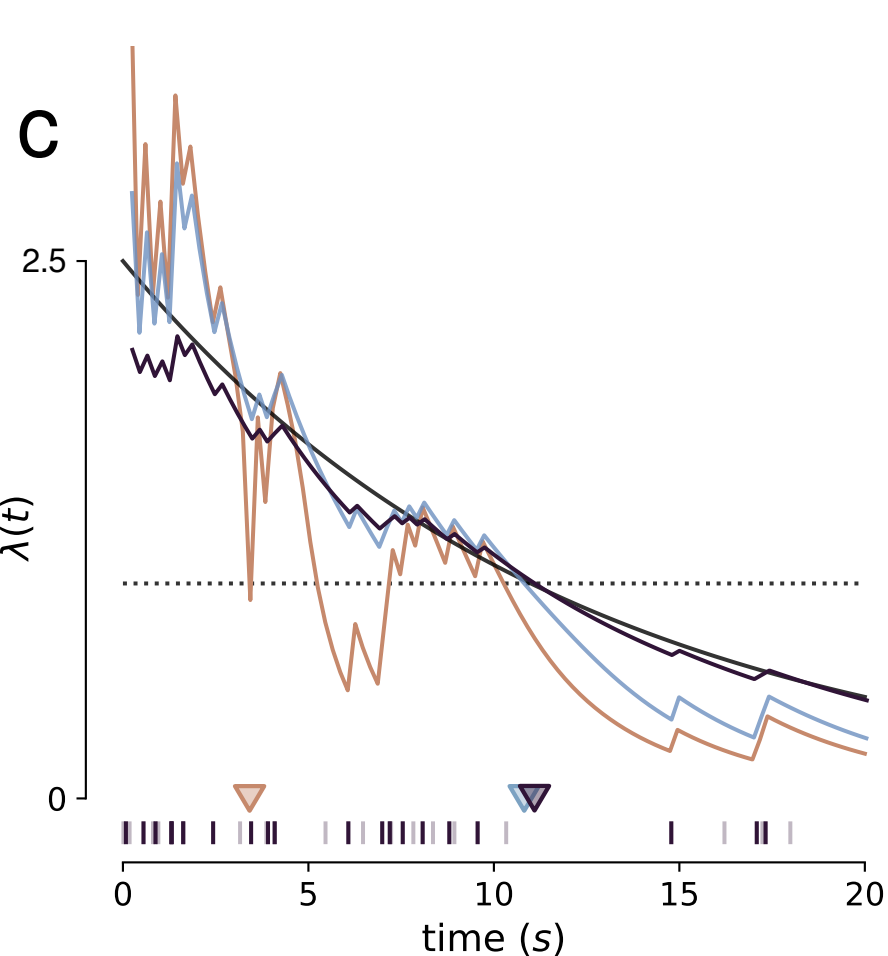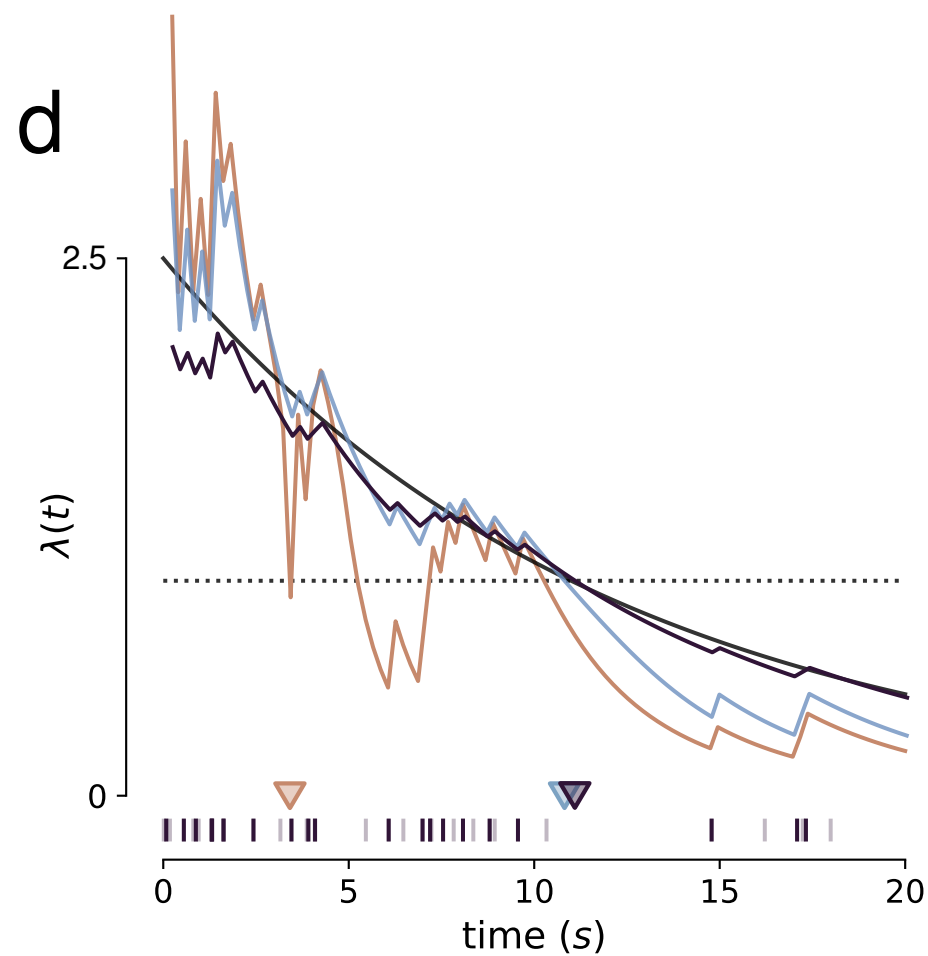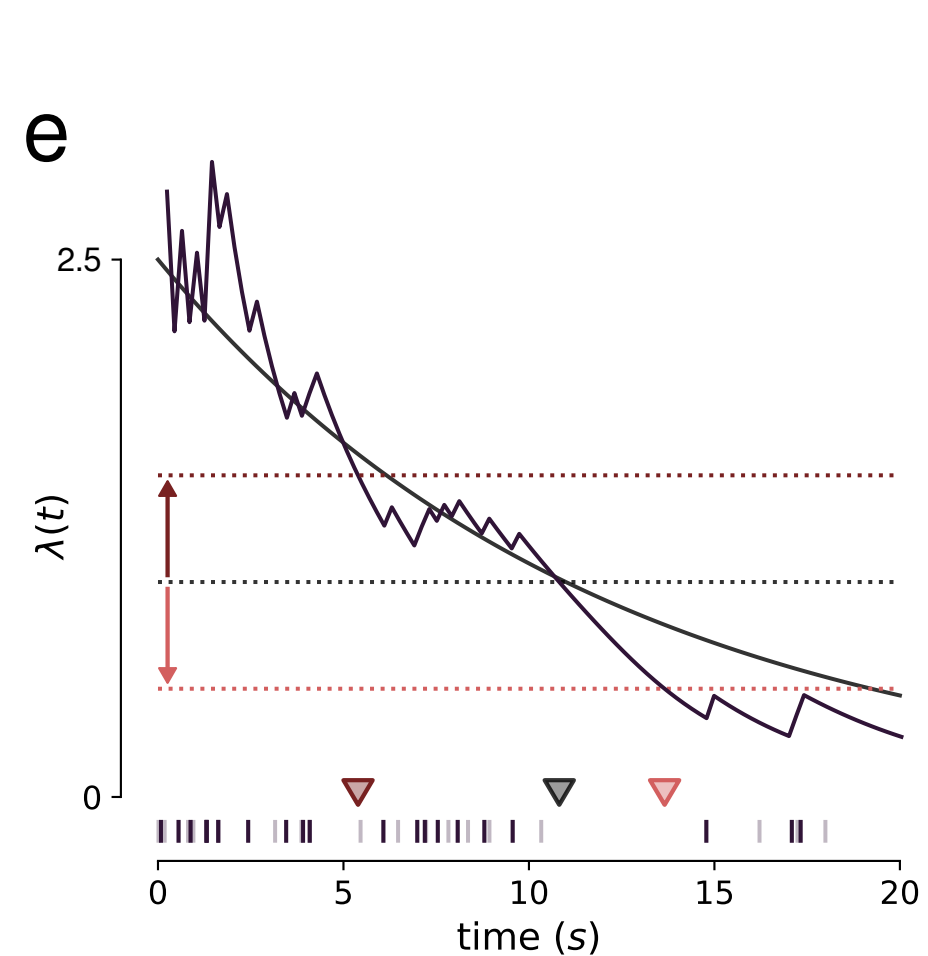

Supplement: S3 Fig — (A) The root-mean-square prediction errors (RMSE) of various MLE-x models (solid line) were calculated for all patches in environments with moderate-to-high stochasticity (RSI∈[0.5,1.0]). In order to estimate the Poisson rate, models utilized the observations from the current patch plus zero (MLE-1) up to ten (MLE-11) of the previous patch encounters. The prediction error asymptotically approached that of the MVT-IM model (dotted line). (B) Two example prior distributions (solid or dotted curve) for the initial Poisson rate (lambda0; left) and decay rate (τ; right) are shown for three different levels of variance (var(p)). Prior distributions were generated from a gamma distribution such that the mode was equal to either the experimental (λ0) or internally-modeled (τ) value for the environment (solid or dotted triangle). (C) The Poisson rate estimates of the MAP-IM-L model are shown for an example reward sequence in a patch (raster at bottom), which consists of unobserved (light purple) and observed (dark purple) events. The rate estimates utilized observations from the example patch and reflect prior distributions with high (orange), moderate (light blue), and low (dark purple) levels of uncertainty, as shown in B. The predicted leaving times for the models (colored triangles) occur when the estimated rates fall below a given threshold for the environment (dotted black line) that is derived from the MVT-IM model. (D) Given the same example reward sequence as in C, the MAP-IM-GL model estimates the Poisson rate (purple solid line) from observations (raster at bottom) and additionally modulates the rate threshold for patch-leaving (black dotted line). Higher (dark red) or lower (light red) thresholds lead to earlier or later leaving times, respectively. The true Poisson rate in C and D is shown by the black curve. (PDF) [file pcbi.1012989.s003.pdf]

**a**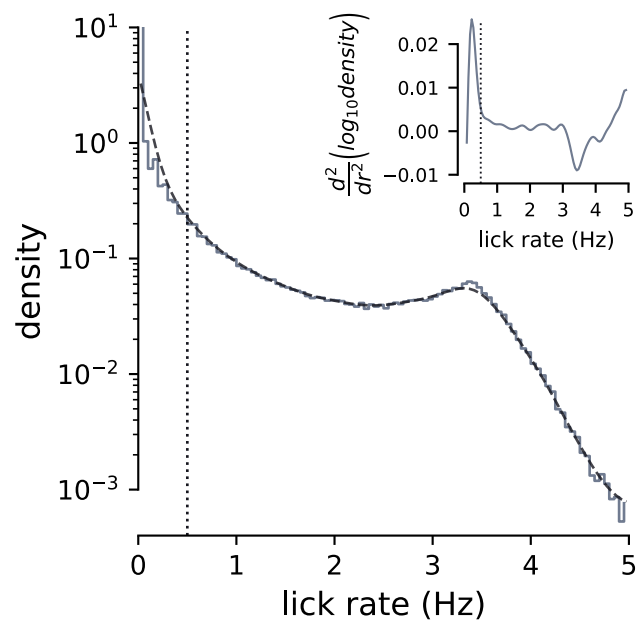**b**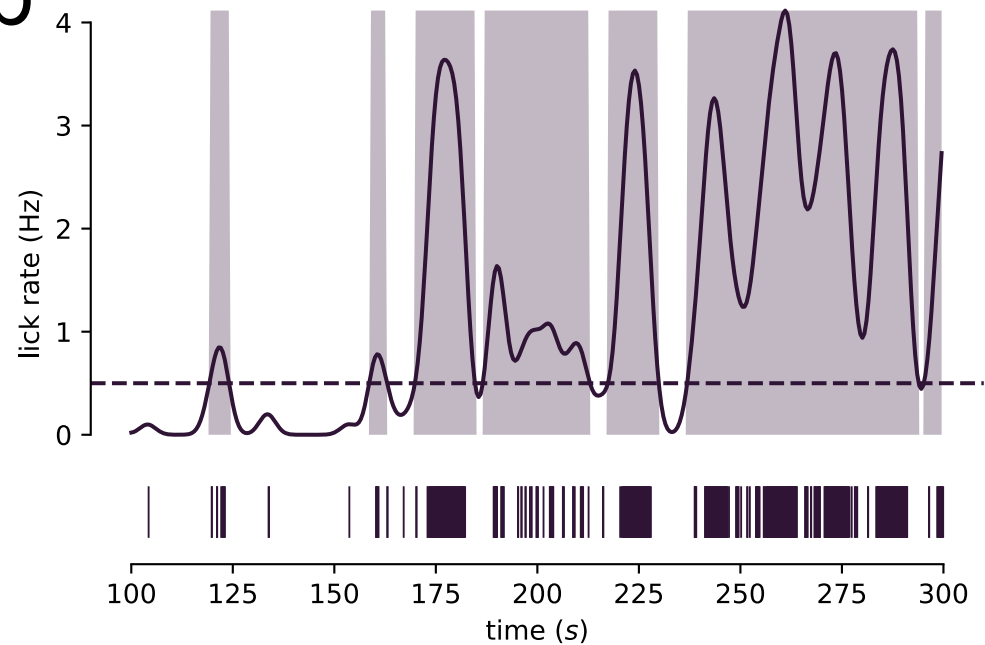**c**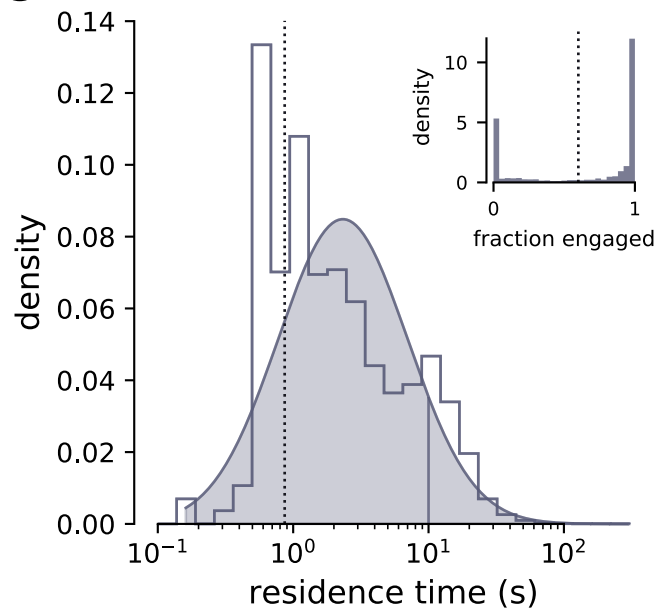**d**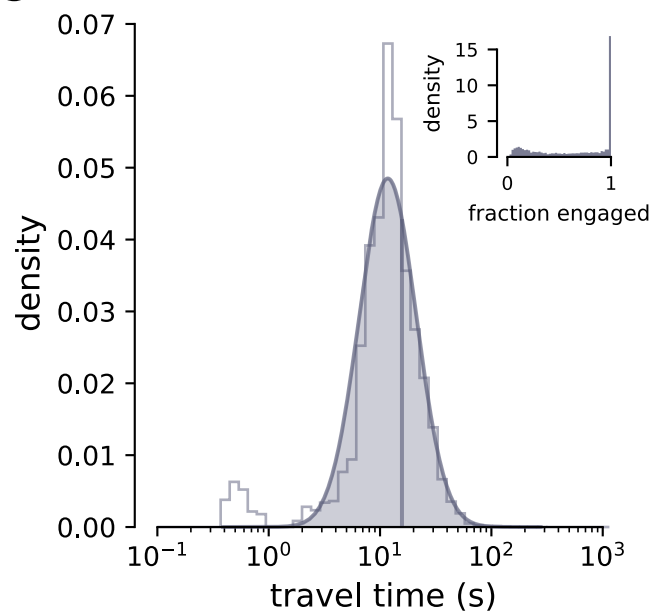**e**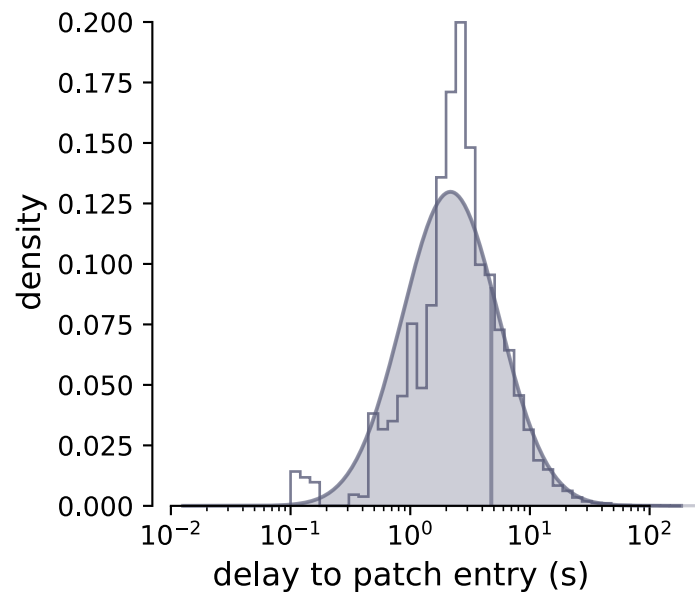

Supplement: S4 Fig — (A) The raw (solid line) and smoothed (dotted curve) histogram of the smoothed lick rate (bin size = 0.5 seconds, σ = 2 seconds) is shown for data pooled from all animals (N = 3) on the head-fixed task. The rate threshold for active engagement (vertical dotted line) was chosen to represent the “elbow" of the second derivative of the smoothed histogram of lick rates (inset). (B) The animal licks (raster at bottom) and smoothed lick rate (solid purple curve) are shown for a 200-second window of an example session. The residence time (shaded areas) was estimated as the time during which the smoothed lick rate exceeded the rate threshold (horizontal dotted line). (C- E) A histogram (step-wise solid line) and fitted log-normal distribution (shaded area) with its associated median (vertical solid line) are shown for the estimated residence times (C; calculated per B), travel times (D; calculated as time between patches during which velocity exceeded 0.5 cm/s), and delay from traveling the required distance to stopping within the next patch (E). (insets) Histograms (purple bins, left axis) and cumulative summations (solid line, right axis) of the fraction of raw residence (C) and travel (D) times during which the animal met the respective engagement criteria. Residence times additionally required at least 60% engagement (vertical dotted line in C) for the patches to be included in the analysis. (PDF) [file pcbi.1012989.s004.pdf]

environment

animal

session

patch

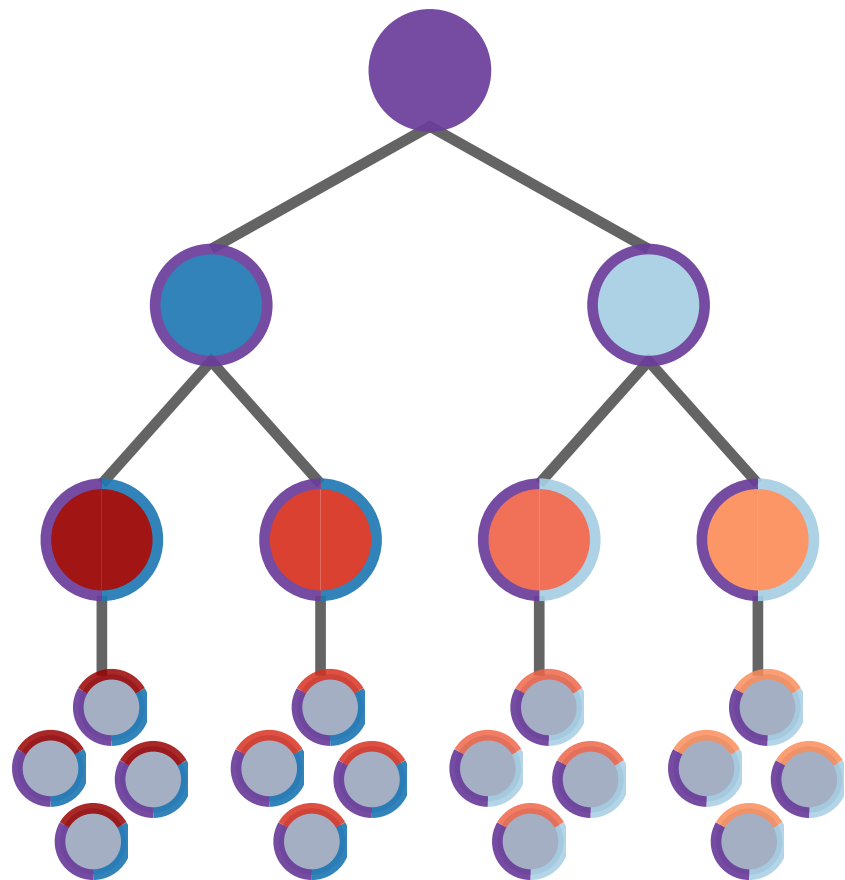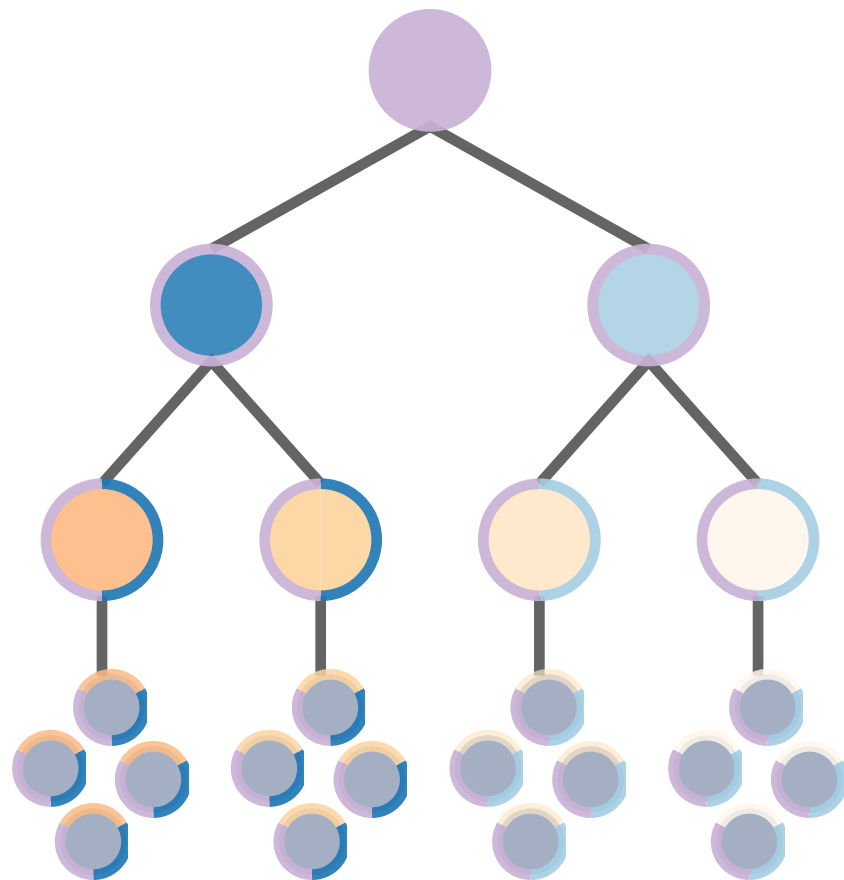

Supplement: S5 Fig — Residence times, which are the data points of analysis, exist within a hierarchical structure of contextual characteristics that influence outcomes, including the environment (such as travel distance or reward decay rate), animal, or session in which the patch occurred. When sampling via the bootstrap method, these characteristics (colored outlines of circles) must be appropriately randomized at each level to respect their individual contributions to the overall outcomes. (PDF) [file pcbi.1012989.s005.pdf]

**a**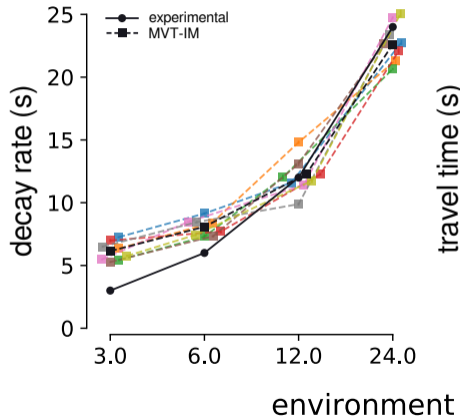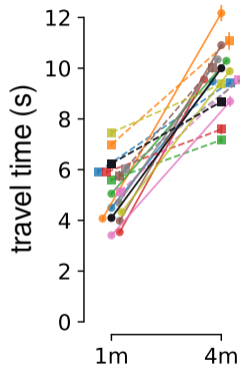**b**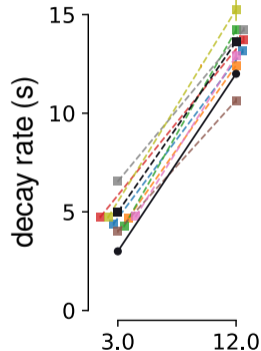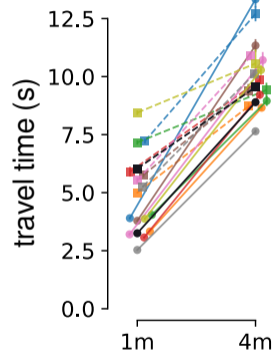

Supplement: S6 Fig — For environments with low- (A; RSI = 0.1) and moderate-to-high (B; RSI∈[0.5,1.0]) stochasticity, the estimates of the reward decay rate (left) and travel time (right) were calculated per the MVT-IM model, which constrained each animal to have a one-to-one mapping between parameter estimates (vertical axis) and unique environments (horizontal axis). Internal estimates are shown for individual animals (colored squares and dotted lines), which were used in the analysis, and for pooled data (black squares and dotted lines), which are shown for visualization purposes. The experimental values are also shown for individual animals (colored circles and solid lines) and pooled data (black circles and solid lines); note that individual experimental decay rates were equivalent (i.e. independent of animal behavior) and thus are not shown. (PDF) [file pcbi.1012989.s006.pdf]

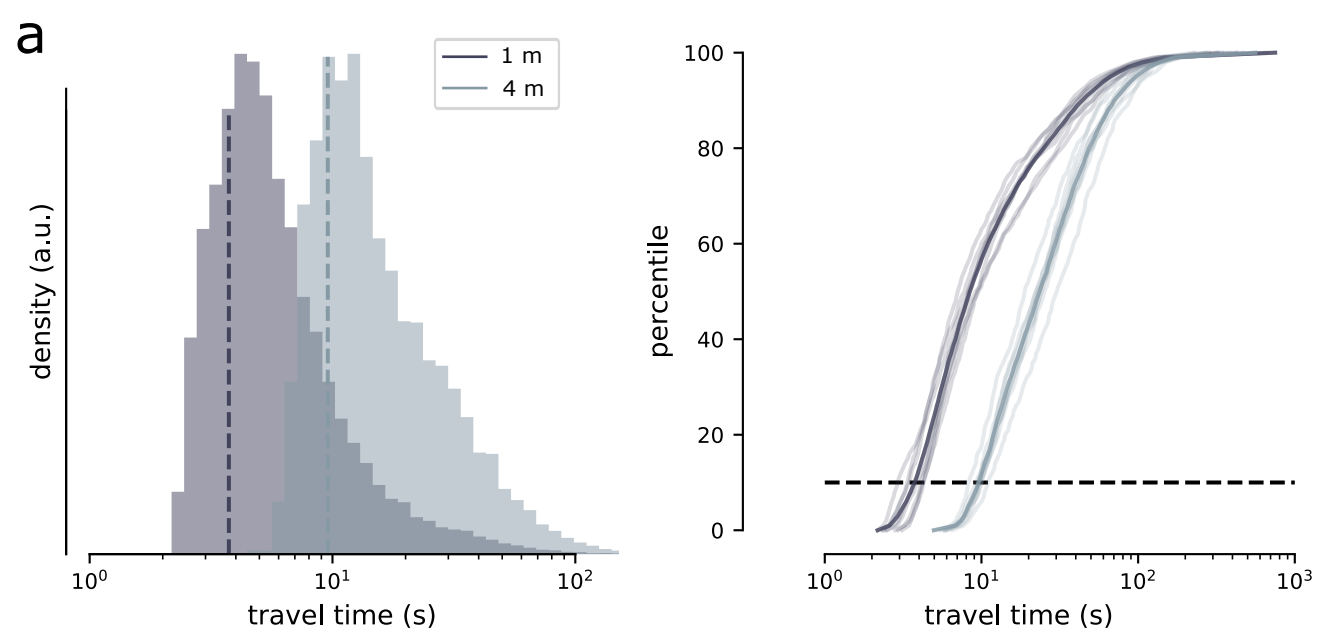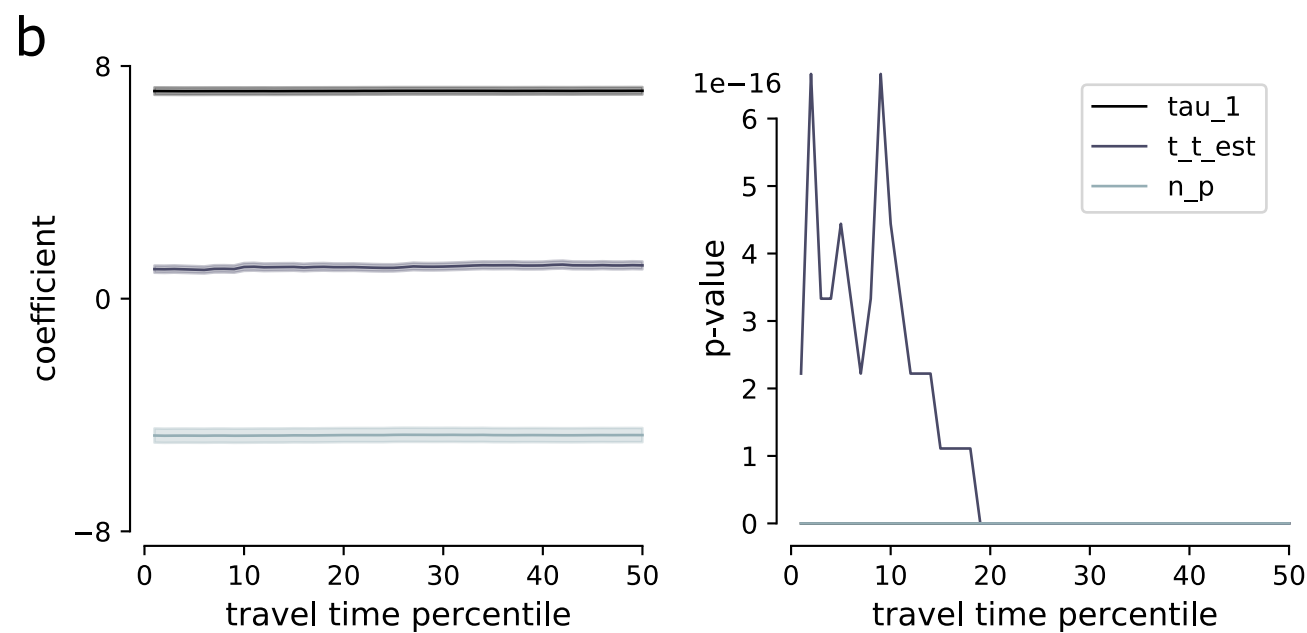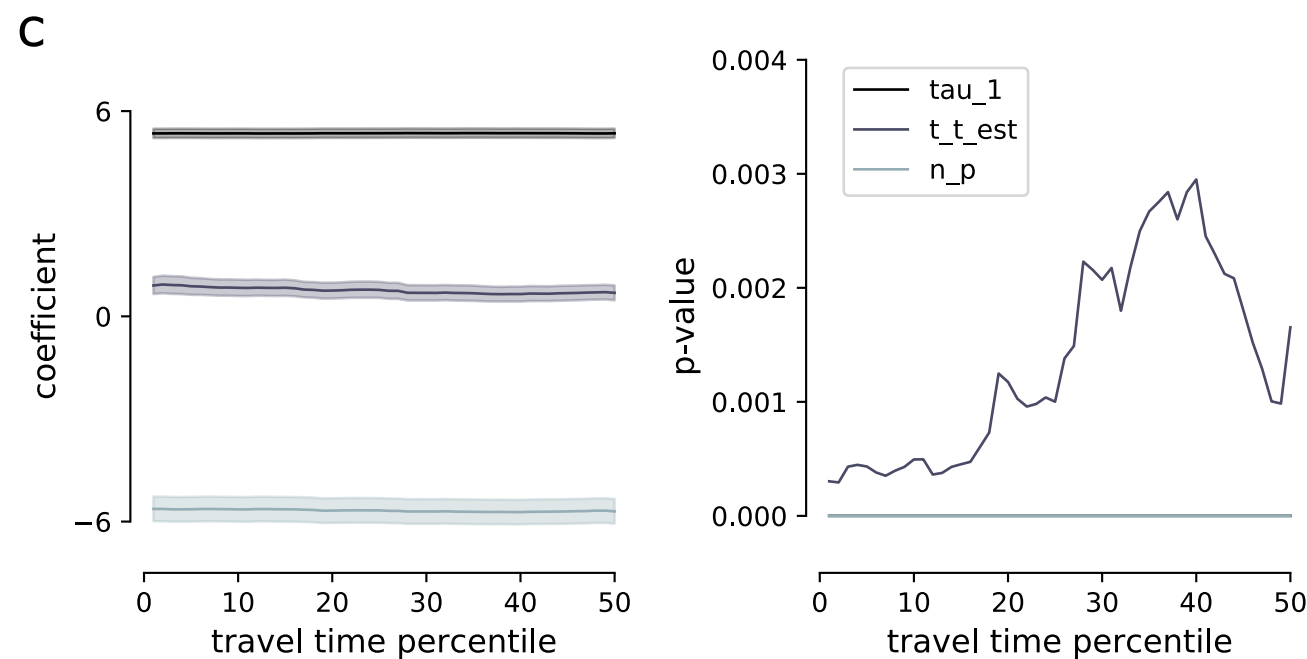

Supplement: S7 Fig — (A) The histogram (left) and cumulative distribution (right) of total travel times (defined as the duration between nose-pokes at successive reward ports) across all freely-moving sessions included in the analysis, categorized by track type (1 meter: purple; 4 meters: light blue). The tenth percentiles of the distributions are represented by the dotted lines in both panels. (B, C) The coefficient values (left) of the linear mixed model fit to data in the low- (B) and high- (C) stochasticity environments are shown for the decay constant (tau; black), task-relevant travel time (t_t_est; purple), and patch number (n_p; light blue). Solid lines and shaded areas represent the mean and 95% confidence intervals, respectively. The corresponding p-value for the model fits are shown at right. Note that the p-values for the decay constant and patch number remain highly significant and overlap for the entirety of the percentile domain. (PDF) [file pcbi.1012989.s007.pdf]
