# Supplementary material for: Foraging animals use dynamic Bayesian updating to model meta-uncertainty in environment representations
Source: PLoS Comput Biol. 2025 Apr 30;21(4):e1012989. doi: 10.1371/journal.pcbi.1012989 (PMC12068741; doi:10.1371/journal.pcbi.1012989)
Supplement: S1 Table — (A) Linear mixed-effects models of the freely moving task. Parameters were fit as predictors of residence time for patches in low-stochasticity (RSI = 0.05, m = 9547 patches) or high-stochasticity (RSI∈[1.0,2.0], m = 4513 patches) environments. All fixed effects were normalized to the range [0,1]. Coefficient values are provided as mean [95% CI]. χ2 and p values were generated from likelihood ratio tests between the full model and reduced model with the respective parameter removed. Key: τ = decay rate, t^(t) = task-relevant travel time, np = patch number. (B) Linear mixed-effects models of the head-fixed task. Notation and analysis follows (A). Low-stochasticity (RSI = 0.05, m = 1299 patches) and high-stochasticity (RSI∈[1.0,2.0], m = 787 patches) environments were analyzed separately. (C) List of environmental parameters. Reward stochasticity index (RSI) and decay rate are defined elsewhere. Decay rate is given in seconds and track length in meters. (PDF) [file pcbi.1012989.s008.pdf]

| RSI | parameter | $\beta$ | | $\chi^2$ | $p$ |
|---|---|---|---|---|---|
| 0.05 | $\tau$ | 7.14 | [ 6.88 , 7.39 ] | 2649.38 | <0.001 |
| | $\hat{t}^{(t)}$ | 1.10 | [ 0.84 , 1.36 ] | 67.13 | <0.001 |
| | $n_p$ | −4.71 | [−5.18 , −4.23 ] | 366.52 | <0.001 |
| 1.0, 2.0 | $\tau$ | 5.35 | [ 5.08 , 5.61 ] | 1354.05 | <0.001 |
| | $\hat{t}^{(t)}$ | 0.84 | [ 0.36 , 1.31 ] | 12.07 | <0.001 |
| | $n_p$ | −5.65 | [−6.35 , −4.94 ] | 238.01 | <0.001 |

Table A: Linear mixed-effects models of the freely moving task.

| RSI | parameter | $\beta$ | | $\chi^2$ | $p$ |
|---|---|---|---|---|---|
| 0.05 | $\tau$ | 8.49 | [ 7.32 , 9.65 ] | 188.67 | <0.001 |
| | $\hat{t}^{(t)}$ | −1.30 | [−2.71 , 0.11 ] | 3.24 | 0.072 |
| | $n_p$ | −3.03 | [−5.06 , −1.00 ] | 7.50 | 0.006 |
| 1.0, 2.0 | $\tau$ | 1.53 | [ 0.47 , 2.58 ] | 7.95 | 0.005 |
| | $\hat{t}^{(t)}$ | 6.30 | [ 4.73 , 7.88 ] | 59.11 | <0.001 |
| | $n_p$ | −2.90 | [−5.23 , −0.56 ] | 5.89 | 0.015 |

Table B: Linear mixed-effects models of the head-fixed task.

| task | RSI | decay rate ($\tau$) | | | | track length | | |
|---|---|---|---|---|---|---|---|---|
| freely-moving | 0.05 | 3 | 6 | 12 | 24 | | 1.0 | 4.0 |
| | 0.50 | 3 | | 12 | | | 1.0 | 4.0 |
| | 1.00 | 3 | | 12 | | | 1.0 | 4.0 |
| head-fixed | 0.05 | 3 | 6 | 12 | | 0.6 | 1.0 | 2.0 |
| | 0.50 | 3 | | 12 | | | 1.0 | 2.0 |
| | 1.00 | 3 | | 12 | | | 1.0 | 2.0 |

Table C: List of environmental parameters.
