# Supplementary material for: Foraging animals use dynamic Bayesian updating to model meta-uncertainty in environment representations
Source: PLoS Comput Biol. 2025 Apr 30;21(4):e1012989. doi: 10.1371/journal.pcbi.1012989 (PMC12068741; doi:10.1371/journal.pcbi.1012989)
Supplement: Attachment — Submitted filename: author_response.docx [file pcbi.1012989.s010.docx]

Dear Editor and Reviewers,

Thank you for your time in reviewing our manuscript for suitability in *PLOS Computational Biology*. We are very grateful for your thorough comments and insights into our work. We have addressed your concerns about 1) the context of our foraging model within previous literature by adding an additional paragraph to the Discussion, and 2) the inclusion/exclusion criteria by further elaborating their reasoning and, for the estimated task-relevant travel time, performing additional analyses. Please find a detailed point-by-point response to your comments and concerns below.

**Reviewer #1**

Main Comments:

1. My main comment is that the contribution of this work does not come across in the Discussion as clearly as it could. Substantial effort was made to explore a wide variety of decision-making mechanisms underlying decision-making but the significance of these results is not thoroughly discussed or placed within the context of previous studies of these topics, especially within the area of behavioral ecology. I suspect that this is due to the authors’ interest in future applications of these methods to better understand neurophysiological processes in mice. However, I think that failing to more thoroughly discuss key findings from the current study does a disservice to these interesting results.

We greatly appreciate your comments and have added additional paragraphs to the Introduction and Discussion, as well as modified the Abstract, to further elaborate on the distinction of these results compared to previous foraging models.

Minor Comments:

1. The authors do not discuss why acoustic cues indicating reward availability are provided to the mice. These acoustic cues represent a fundamental difference between the presented experimental set-up and natural environments, particularly for species that do not have access to visual information on patch quality and must directly sample a patch to determine its profitability. I would like to see some discussion of the authors’ choice to use these cues in their experimental design.

The stochastic distribution of the rewards, driven by an inhomogeneous gamma process, was itself motivated by naturalistic sampling (see e.g. Kilpatrick et al., 2020). Thus we wanted animals to observe rewards in a manner consistent with this distribution to build their behavioral models. Allowing animals in this case to dictate the timing of sampling, and thus observations, would obfuscate the distribution from which the rewards were generated and make it unclear what, if any, distribution underlay the reward-generating process. This became especially important for the head-fixed task because animals licked more sparsely compared to the freely-moving task. To this end, sounds provided the rapid and experimentally controllable information that was needed for an accurate estimation of the underlying reward distribution. We have added a clarifying sentence under the Results subsection, *A patch-based foraging task in freely moving mice*.

2. In the Figure 2 legend, I believe the first word should be “Mice” rather than “Match”

Typo fixed, thank you.

3. In the Inclusion and Exclusion subsection of the Methods, the authors describe two different processes that they use for identifying outliers that differ between data collected from freely-moving mice and head-fixed mice. Specifically, in freely-moving mice, outliers are defined as being more than three standard deviations above or below the mean whereas in head-fixed mice, outliers are defined as being two standard deviations below the mean. I ask that the authors please provide justification for these differences.

Compared to those in the freely moving task, the log-normal distribution of estimated residence times for the head-fixed task was skewed towards shorter time scales. Selecting two instead of three standard deviations for the threshold allowed us to avoid unreasonably small residence times in our analyses and provide a more conservative estimate of behavioral changes amongst the different environments. We have added a sentence in the Methods section, under the subsection Inclusion and Exclusion Criteria.

**Reviewer #2**

Main Comments:

1. What age were the mice? This is important because recent work suggests that exploration strategies in standard reward tasks and also more simply structured foraging tasks vary noticeably with development stage (in humans, NHP, and mice — see e.g. Johnson & Wilbrecht 2011, Dev Cog Neuro), and also because it would be valuable to understand whether the feasibility of training mice in this task at early developmental stages has been established. I apologize if this was reported somewhere and I missed it—if so, please copy the report into the ‘Animals’ subsection of Methods and Materials.

We agree that this information is important and have updated the Animals subsection accordingly.

2. The decision to exclude the tenth percentile of travel time is reasonable, but raises the question of how robust the results are to this somewhat arbitrary thresholding. Can the authors provide evidence, up to and perhaps including if necessary a repeat of the model-based analyses at different values of this threshold? Of course, if the results are in fact sensitive to this thresholding, it would not be a reason to discount the results of the study; rather, it would provide valuable information for future researchers who wish to adopt this task and associated models.

The tenth percentile approximated the inflection point of the cumulative distribution of total travel times and thus, in a simple model that differentiates between shorter task-relevant and longer task-irrelevant travel times, estimates the boundary between the two. However, we investigated the sensitivity of the results to this threshold as you suggested and found that the results were robust across a wide range of values. We have added our findings in Supplementary Figure 7 and made a brief note in paragraph 2 of the Results subsection, *Mice adapt their behavior to daily perturbations in the environmental statistics*, as well as paragraph 1 of the Methods subsection, *Residence and Travel Times*.

3. Relatedly, can the authors more finely characterize the behaviors performed during these excluded transits? Grooming is clear, but (as mentioned in the Discussion section) ‘exploring’ may refer to several different kinds of behavior, some of which may be relevant for inferring mechanisms—e.g. deliberation, “vicarious trial and error”-like head swivels, changes of mind, etc. Of course, this may be difficult or infeasible given the large number of transits involved—a broad characterization of a subset of qualitatively distinct behaviors would be sufficient

In addition to grooming, animals sometimes “explored” the features of the physical apparatus, namely the walls. This behavior manifested as rearing, sniffing, and occasionally attempting to climb some of the connecting hardware. There were some instances of more deliberative behaviors near the reward port, such as quick head swivels and changes of mind while nose-poked. However, the time scale of these were much shorter (less than one second), which is why we required un-poking to be more than 500 ms prior to accepting it as patch-leaving. We have added a sentence in the second-to-last paragraph of the Discussion to elaborate on this point.

4. Of particular interest is whether these longer travel times might in some cases be consistent with a model in which the mice are attempting to harvest information about potentially multimodal reward distributions, rather than responding to a higher local reward stochasticity alone [see e.g. Harhen & Bornstein 2022, 2023 in humans and for an explicit implementation of this model; see also concordant findings by Garcia, Gupta, Wikenheiser 2023 in rats; Barack et al 2024 in humans and monkeys]. It’s difficult to tell whether the data support this idea, though a motivated inspection of Fig. 4B,C may suggest some multimodality in residence time deviations from the average. Of course, a key difference in this design is the use of perfectly unpredictable transitions between patch types, which may frustrate animals’ attempts to infer distinct reward modes. However, a response to this question might help inform future designs that more closely examine this question.

The existence of multimodality in animal behavior is an excellent point and one of keen interest to our foraging models. However, our interpretation of the data is that while possible, the animals likely built a unimodal model that was continuously updated (in conjunction with our response below). In the task structure, the travel distance was fixed, meaning that travel times were entirely a product of animal speed and vigor; thus behavioral decisions reflecting a multimodal model would stem only from deviations in residence time. Moreover, in agreement with your latter point, the reward process likely did not contain sufficient structure on a patch-to-patch basis to allow for interpretation of multiple underlying distributions, which we have added in the fourth paragraph of the Discussion. To explore this idea in future studies, one approach would be to explicitly tailor particular reward ports and/or blocks within sessions to have distinct reward-rate parameters.

5. Is there evidence that local and global reward statistics are continually updated in a manner that reflects ongoing learning consistent with an approximate strategy (see e.g. Wilson et al., 2013, Plos Comp Bio), rather than an exact Bayesian approach whose effective ‘learning rate’ may asymptote with experience? Again, either finding would be consistent with the authors’ overall conclusions—a response to this question is primarily useful for guiding future analysis and research designs.

Yes, the observed behavior supports the former idea, that the decision-making model continually updates based on recent observations rather than accrues evidence over longer timescales for an exact approach. See, for instance, Supplementary Figure 3a. When using only likelihood (MLE-x), the model asymptotes towards the true parameter values as one would expect but does not describe behavior more accurately than the MVT model (MVT-IM). That is, the inherent patch-to-patch deviations that are better matched by a MAP model reflect an ongoing learning rate based on the current reward structure. We have added a sentence in the new Discussion paragraph to more explicitly state this finding.
