# Supplementary material for: Foraging animals use dynamic Bayesian updating to model meta-uncertainty in environment representations
Source: PLoS Comput Biol. 2025 Apr 30;21(4):e1012989. doi: 10.1371/journal.pcbi.1012989 (PMC12068741; doi:10.1371/journal.pcbi.1012989)
Supplement: Attachment — Submitted filename: Reviewer Comments.docx [file pcbi.1012989.s009.docx]

The authors seek to address critical constraints of the marginal value theorem, which are that it assumes that environmental properties (i.e., average food intake rate) are stable and that foragers have a good understanding of those properties. The marginal value theorem has been a dominant framework for analyzing and interpreting animal foraging behavior, often through the use of experiments that test decision-making in relatively simple artificial foraging environments. However, as these environments are often not representative of the variability present in natural foraging environments, decision-making under these more complex foraging conditions is much less understood. The authors develop an experimental framework to simulate greater levels of environmental variability to determine how well the marginal value theorem (and other behavioral proxies) explains behavior under these conditions and whether more flexible decision-making (i.e. adhering to a Bayesian approach) may better explain behavior. An additional aim of the study is to demonstrate how a modification to the developed approach using head-fixed mice could be used to study the neurophysiological mechanisms underlying decision-making in foraging contexts.

Similar to many tests of the marginal value theorem, the authors found that mouse behavior adhered to qualitative predictions of the marginal value theorem in that they remained longer in patches when travel time was longer and when reward decay rate was slower. This was true not only of freely moving mice but also head-fixed mice foraging in a virtual environment, although the effect of travel time was not observed in the low stochasticity environment. In freely-moving mice, the authors also found that the marginal value theorem better predicted mouse behavior than more simple behavioral heuristics, like remaining in a patch until a set number of rewards had been obtained or staying in a patch until a set amount of time had passed since obtaining a reward. However, the heuristic of tracking time since last reward partially explained behavior in the high stochasticity environment.

Uniquely, the authors also compare mouse behavior to a model of the marginal value theorem where environmental parameters are estimated from the data in order to obtain the best fit of the model. This model asks how closely the model might fit behavior if the mice imperfectly perceived environmental parameters. An interesting contribution is that they found that this model best fit the observed behavior but with the caveat that the mice underestimated reward decay rate. The authors also found that variability in patch-leaving behavior within a given session could be explained by variation in perceived decay rate due to stochasticity in reward sequences, with mice remaining longer in patches when the observed reward sequences lead to slower perceived decay rates and vice versa. When considering the impact of both global environmental characteristics and local experience in given foraging patches, the authors find that this combination of both perceived global and local information best explains mouse foraging behavior.

I believe this paper makes a meaningful contribution to both understanding of the cognitive processes underlying foraging decisions as well as the development of methods to study the neurophysiological processes underlying these decisions. The authors present a rigorous experimental and analytical framework for studying and analyzing decision-making. Their study goes beyond most traditional studies by exploring a wider variety of cognitive processes that may underlie foraging decision-making while also examining these processes across multiple levels of environmental stochasticity. My main comment is that the contribution of this work does not come across in the Discussion as clearly as it could. Substantial effort was made to explore a wide variety of decision-making mechanisms underlying decision-making but the significance of these results is not thoroughly discussed or placed within the context of previous studies of these topics, especially within the area of behavioral ecology. I suspect that this is due to the authors’ interest in future applications of these methods to better understand neurophysiological processes in mice. However, I think that failing to more thoroughly discuss key findings from the current study does a disservice to these interesting results.

Minor comments:

The authors do not discuss why acoustic cues indicating reward availability are provided to the mice. These acoustic cues represent a fundamental difference between the presented experimental set-up and natural environments, particularly for species that do not have access to visual information on patch quality and must directly sample a patch to determine its profitability. I would like to see some discussion of the authors’ choice to use these cues in their experimental design.

In the Figure 2 legend, I believe the first word should be “Mice” rather than “Match”

In the Inclusion and Exclusion subsection of the Methods, the authors describe two different processes that they use for identifying outliers that differ between data collected from freely-moving mice and head-fixed mice. Specifically, in freely-moving mice, outliers are defined as being more than three standard deviations above or below the mean whereas in head-fixed mice, outliers are defined as being two standard deviations below the mean. I ask that the authors please provide justification for these differences.
